# Association Between a History of Breast Cancer and Decreased Thyroid Cancer-specific Mortality

**DOI:** 10.1210/clinem/dgad722

**Published:** 2023-12-08

**Authors:** Shuhuang Lin, Zhuo Wang, Mingzhao Xing

**Affiliations:** Thyroid Research Institute, School of Medicine, Southern University of Science and Technology, Shenzhen, Guangdong 518055, China; Thyroid Research Institute, School of Medicine, Southern University of Science and Technology, Shenzhen, Guangdong 518055, China; Thyroid Research Institute, School of Medicine, Southern University of Science and Technology, Shenzhen, Guangdong 518055, China

**Keywords:** thyroid cancer, breast cancer, mortality, prognosis

## Abstract

**Context:**

The clinical relevance of the well-known association between thyroid cancer (TC) and breast cancer (BC) remains to be further defined.

**Objective:**

This work aimed to investigate the effect of history of BC on the prognosis of TC.

**Methods:**

This was a comparative cohort study of tumor behaviors and TC-specific mortality in 5598 patients with papillary thyroid cancer (PTC) and 604 patients with follicular thyroid cancer (FTC), all with a history of BC (TC-BC patients), and their propensity score–matched TC patients without a history of BC (TCnoBC patients) in Surveillance, Epidemiology and End Results (SEER) 18. The main outcome measure was TC-specific mortality.

**Results:**

Lower TC distant metastasis rates of 2.4% vs 3.0% in PTC and 6.1% vs 9.1% in FTC and TC-specific mortality rates of 1.3% vs 2.6% in PTC and 5.8% vs 8.4% in FTC were found in TC-BC patients vs matched TCnoBC patients (all *P* < .05). Comparing TC-BC patients with matched TCnoBC patients, hazard ratios (HRs) for mortality were 0.472 (95% CI, 0.370-0.601) in PTC and 0.656 (95% CI, 0.461-0.934) in FTC (all *P* < .05). Such HRs for mortality in PTC were 0.397 (95% CI, 0.268-0.588; *P* < .001) when TC occurred before BC vs 0.607 (95% CI, 0.445-0.827; *P* = .002) when BC occurred before TC.

**Conclusion:**

This study demonstrates a robust protective effect of a history of BC on TC-specific patient survival, which has strong implications for more precise prognostication of TC in such patients.

Thyroid cancer (TC) is a common endocrine malignancy with a rapid increase in incidence in the United States and globally in recent decades (1-3). Papillary thyroid cancer (PTC) and follicular thyroid cancer (FTC) account for about 90% and 10% of cases of TC, respectively, and 75% to 80% of patients are women (4, 5). Like TC, breast cancer (BC), another endocrine-related malignancy, is also common in women (6). There is an established association between TC and BC (4, 7, 8). Patients with an elevated risk of 55% and 18% were estimated to develop a TC after a BC and to develop a BC after a TC, respectively, compared with the general population (9). The mortality of BC has substantially decreased in recent decades due to improved treatments, including surgical and endocrine therapies, based on its molecular classification (10, 11). As a result, there are now increasing cases of survivors with a history both of TC and BC (4, 6).

A history of TC was shown to be associated with decreased BC-specific mortality (12). It remains to be established whether a history of BC may, inversely, affect the clinical outcomes of TC. We tested this possibility by examining the effect of a history of BC on the clinical prognosis, particularly mortality risk, of TC using Surveillance, Epidemiology, and End Results (SEER) 18.

## Materials and Methods

### Data Sources

With permission, we used the SEER 18 database (November, 2018 submission) (https://seer.cancer.gov) combined with the US County Population Data (1969-2019) for the present study. A detailed description of the two databases can be seen in the Supplementary Materials I (13). We collected records of TC diagnosed since January 1, 1983, the time when SEER started collecting detailed information of TC.

### Cohort Definition

Only female patients were included in this study. Patients with differentiated TC and BC in SEER 18 were identified using codes of the International Classification of Diseases for Oncology, third revision (ICD-O-3) (14) (Supplementary Materials I (13)). TC or BC found at death or autopsy was excluded in this study (15).

For convenient presentation in this study, patients with TC were designated as TC-BC if they also had a history of BC and TCnoBC if they had no history of BC. Based on the diagnosis sequence, TC-BC patients were further grouped into those who had the diagnosis of TC first (TC-1st) and those who had the diagnosis of BC first (BC-1st).

We used the following strategies to minimize participant selection bias between TC-BC/TC-1st/BC-1st and their matched TCnoBC groups. Patients who died of BC were excluded so patients in the TC-BC/TC-1st/BC-1st groups were not affected by the BC-specific mortality as in the TCnoBC group. Moreover, TCnoBC patients were selected to correspondingly match TC-BC, TC-1st, or BC-1st patients for age at the diagnosis of TC and TC incidence densities in the same year, to reduce the influence of patient age and the changes in the treatment strategy for TC over the years. This step was achieved using a propensity score matching approach with a TC-BC/TC-1st/BC-1st to TCnoBC ratio of 1 : 5 (16).

### Variable Selection and Outcome Measures

Clinicopathological characteristics and patient survival of TC were treated as the main outcome measures in this study. The clinicopathological characteristics included patient age at diagnosis, pathological histology, combined SEER stage, tumor size, and status of tumor extension, lymph node (LN) metastasis, distant metastasis, and multifocality as described in the supplementary materials (13). For the analysis of survival and follow-up, we extracted data on the cause of death and vital status for each TC patient. Survival time was estimated by subtracting the date of the diagnosis of TC from the date of patient death or, in case of no death, the date of last contact.

The demographic and socioeconomic status (SES) information was extracted for controlling potential confounding effects, with the former including race/ethnicity and state/county and the latter including marital status, medical insurance, employment status, education level (at least high school education), and median family income. Among them, race/ethnicity and state/county, marital status, and medical insurance were at the individual level for each patient, while education level, unemployment rate and family income were evaluated at the county level by census. Data on medical insurance were available after 2007 and data on education, unemployment, and family income were available after 1990. The data on estrogen receptor (ER) and progesterone receptor (PR) were available after 1990 and the data on human epidermal growth factor (HER2) were available after 2010.

Information on some variables was not available (NA) for some patients. The distribution and pattern of the NA data are shown in Supplementary Fig. S1 in the supplementary materials (13). The NA data were filled using multiple imputation with the random forest method (17). For medical insurance, education level, unemployment rate, and family income in SES, and ER, PR, HER2 status, as well as BC subtypes in BC characteristics, the NA data were similarly filled.

### Statistical Analysis

Categorical data were summarized as frequencies and percentages. Continuous data, which were not normally distributed in this study, were summarized as medians and interquartile ranges (IQR). The χ^2^ test was used to analyze categorical variables. The Wilcoxon-Mann-Whitney test was used to analyze continuous variables. The life-table method was used to determine cumulative mortality (CM). Competitive risk model were used to estimate the TC-specific CM (TSCM). Competitive risk events were derived from causes of death unrelated to TC. Log-rank test was used to construct Kaplan-Meier (K-M) survival curves. Cox proportional-hazard regression analysis was used to adjust covariates and examine hazard ratios (HRs) of the effect of a history of BC on TC-specific mortality. All *P* values were 2-tailed and a *P* less than .05 was considered statistically significant. Statistical analyses were performed using R (version 4.0.3) and its associated packages (17, 18).

## Results

### Relationship Between Clinicopathological Characteristics of Thyroid Cancer and History of Breast Cancer

Comparisons of the clinicopathological characteristics of TC between patients with and without a history of BC are summarized in Tables 1 and 2. In comparison with the matched TCnoBC cases in the corresponding groups, PTC or FTC in patients with a history of BC had a lower TC-specific mortality, with TC-BC vs TCnoBC being 1.3% vs 2.6% in PTC (*P* < .001) and being 5.8% vs 8.4% in FTC (*P* = .035). The difference of TC-specific mortality between TC-1st and matched TCnoBC was even more dramatic, being 1.1% vs 2.5% in PTC and 4.1% vs 8.3% in FTC, respectively (all *P* < .05). In patients whose TC was diagnosed after BC (BC-1st), PTC also showed a reduced TC-specific mortality, with BC-1st vs TCnoBC being 1.5% vs 2.4% (*P* = .002), but FTC did not (*P* = .726); the latter had a relatively small number of patients, though.

In addition to TC-specific mortality, less aggressive TC tumor characteristics in patients with a history of BC were also observed (Table 1). For example, compared with TCnoBC patients, PTC in TC-BC patients showed smaller tumor sizes (*P* < .001) and less tumor extension beyond capsule or further extension (*P* = .02), regardless of the diagnosis sequence of TC and BC. PTC in TC-BC patients also had a lower rate of distant metastasis compared with TCnoBC patients, being 2.4% vs 3.0% (*P* = .024), which is the strongest prognostic predictor for PTC-specific mortality.

**Table 1.** Comparison of clinicopathological characteristics of papillary thyroid cancer in various clinical settings

Variable	Level	Comparison 1			Comparison 2			Comparison 3		
		TC-BC	TCnoBC	*P*	TC-1st	TCnoBC	*P*	BC-1st	TCnoBC	*P*
n		5598	27 990		2571	12 855		3027	15 135	
Age, y (median [IQR])		57.00 (48.00-67.00)	57.00 (48.00-67.00)	.844	53.00 (44.00-63.00)	53.00 (44.00-63.00)	.856	61.00 (52.00-69.00)	61.00 (52.00-69.00)	.835
Race (%)	White	4572 (81.7)	23 073 (82.4)	.012*^c^*	2124 (82.6)	10 739 (83.5)	.684	2448 (80.9)	12 305 (81.3)	.03*^c^*
	Black	371 (6.6)	1729 (6.2)		157 (6.1)	723 (5.6)		214 (7.1)	950 (6.3)	
	Other	645 (11.5)	3172 (11.3)		286 (11.1)	1375 (10.7)		359 (11.9)	1871 (12.4)	
Pathological subtype (%)	CPTC	3744 (66.9)	18 589 (66.4)	.793	1707 (66.4)	8704 (67.7)	.321	2037 (67.3)	9861 (65.2)	.057*^d^*
	FVPTC	1784 (31.9)	9049 (32.3)		844 (32.8)	4036 (31.4)		940 (31.1)	5038 (33.3)	
	Others	70 (1.3)	352 (1.3)		20 (0.8)	115 (0.9)		50 (1.7)	236 (1.6)	
Stage (%)	Localized	4032 (72.0)	19 900 (71.1)	.056	1822 (70.9)	9009 (70.1)	.226	2210 (73.0)	10 845 (71.7)	.154
	Regional	1430 (25.5)	7255 (25.9)		695 (27.0)	3502 (27.2)		735 (24.3)	3796 (25.1)	
	Distant	136 (2.4)	835 (3.0)		54 (2.1)	344 (2.7)		82 (2.7)	494 (3.3)	
Tumor size, cm (%)	<1	2204 (39.4)	10 690 (38.2)	<.001*^a^*	956 (37.2)	4673 (36.4)	.57	1248 (41.2)	6005 (39.7)	<.001*^a^*
	1-2	2061 (36.8)	9835 (35.1)		913 (35.5)	4604 (35.8)		1148 (37.9)	5253 (34.7)	
	2-4	1040 (18.6)	5651 (20.2)		559 (21.7)	2778 (21.6)		481 (15.9)	2842 (18.8)	
	>4	293 (5.2)	1814 (6.5)		143 (5.6)	800 (6.2)		150 (5.0)	1035 (6.8)	
Extension (%)	Localized	4692 (83.8)	23 103 (82.5)	.02*^c^*	2195 (85.4)	10 777 (83.8)	.095*^d^*	2497 (82.5)	12 258 (81.0)	.04*^c^*
	Beyond capsule	874 (15.6)	4655 (16.6)		360 (14.0)	1964 (15.3)		514 (17.0)	2743 (18.1)	
	Further	32 (0.6)	232 (0.8)		16 (0.6)	114 (0.9)		16 (0.5)	134 (0.9)	
LN Met (%)	Yes	958 (17.1)	4991 (17.8)	.206	468 (18.2)	2397 (18.6)	.617	490 (16.2)	2623 (17.3)	.134
	No	4640 (82.9)	22 999 (82.2)		2103 (81.8)	10 458 (81.4)		2537 (83.8)	12 512 (82.7)	
Dis Met (%)	Yes	135 (2.4)	833 (3.0)	.024*^c^*	53 (2.1)	344 (2.7)	.084*^d^*	82 (2.7)	494 (3.3)	.125
	No	5463 (97.6)	27 157 (97.0)		2518 (97.9)	12 511 (97.3)		2945 (97.3)	14 641 (96.7)	
Multifocality (%)	Yes	1602 (28.6)	7908 (28.3)	.833	575 (22.4)	2689 (20.9)	.166	1027 (33.9)	5350 (35.3)	.296
	No	2351 (42.0)	11 857 (42.4)		752 (29.2)	3946 (30.7)		1599 (52.8)	7780 (51.4)	
	Not 2004+	1645 (29.4)	8225 (29.4)		1244 (48.4)	6220 (48.4)		401 (13.2)	2005 (13.2)	
TC-specific mortality (%)	Yes	72 (1.3)	733 (2.6)	<.001*^a^*	27 (1.1)	320 (2.5)	<.001*^a^*	45 (1.5)	366 (2.4)	.002*^b^*
	No	5526 (98.7)	27 257 (97.4)		2544 (98.9)	12 535 (97.5)		2982 (98.5)	14 769 (97.6)	
Marital status (%)	Married	3689 (65.9)	18 122 (64.7)	.102	1752 (68.1)	8568 (66.7)	.148	1937 (64.0)	9499 (62.8)	.209
	Unmarried	1909 (34.1)	9868 (35.3)		819 (31.9)	4287 (33.3)		1090 (36.0)	5636 (37.2)	
Insurance (%)	Yes	3190 (57.0)	15 802 (56.5)	.002*^b^*	891 (34.7)	4430 (34.5)	.59	2299 (75.9)	11 359 (75.1)	<.001*^a^*
	No	33 (0.6)	313 (1.1)		16 (0.6)	105 (0.8)		17 (0.6)	221 (1.5)	
	Not 2007+	2375 (42.4)	11 875 (42.4)		1664 (64.7)	8320 (64.7)		711 (23.5)	3555 (23.5)	
Education (median [IQR])		84.51 (79.26-88.81)	84.36 (78.27-88.70)	.003*^b^*	83.55 (78.16-87.47)	83.55 (77.54-87.50)	.342	85.86 (80.09-89.59)	85.39 (79.03-89.29)	.002*^b^*
Income (median [IQR])		5624.00 (4520.00-6524.00)	5624.00 (4495.00-6525.00)	.706	5367.00 (4219.00-6054.00)	5321.00 (4219.00-6033.00)	.465	5800.00 (4912.00-6994.00)	5795.00 (4946.00-7059.00)	.862
Unemployment (median [IQR])		7.80 (5.87-9.02)	7.80 (5.93-9.04)	.274	7.38 (5.50-8.63)	7.51 (5.47-8.50)	.972	7.87 (6.14-9.40)	7.87 (6.35-9.59)	.15

Abbreviations: BC, breast cancer; BC-1st, TC patients diagnosed after BC; CPTC, conventional papillary TC; Dis, distant; FVPTC, follicular-variant papillary TC; IQR, interquartile range; LN, lymph node; Met, metastases; TC, thyroid cancer; TC-1st, TC patients diagnosed before BC; TCnoBC, TC patients without history of BC.

^*a*^ *P* less than .001; *^b^P* less than .01; *^c^P* less than .05; *^d^P* less than .1.

Compared with TCnoBC patients, FTC in TC-BC patients also showed smaller tumor sizes (*P* < .001), less extrathyroidal extension (*P* = .036), and earlier stage (*P* = .04) (Table 2). Moreover, compared with TCnoBC patients, FTC in TC-BC patients had a lower distant metastasis rate, being 6.1% in the latter vs 9.1% in the former (*P* = .02). This pattern was particularly seen in TC-1st patients, in which the FTC tumor had more common localized disease and less common tumor extension and distant metastasis compared with matched TCnoBC patients (all *P* < .05). FTC in BC-1st patients only showed less common occurrence of tumors larger than 2.0 cm (*P* < .001), with no difference in other tumor characteristics compared with FTC in matched TCnoBC patients (all *P* > .05) (see Table 2).

**Table 2.** Comparison of clinicopathological characteristics of follicular thyroid cancer in various clinical settings

Variable	Level	Comparison 1			Comparison 2			Comparison 3		
		TC-BC	TCnoBC	*P*	TC-1st	TCnoBC	*P*	BC-1st	TCnoBC	*P*
n		604	3020		314	1570		290	1450	
Age, y (median [IQR])		61.00 (50.00-71.00)	61.00 (50.00-70.00)	.743	55.00 (45.00-66.00)	55.00 (45.00-66.00)	.848	67.00 (57.00-74.00)	66.00 (57.00-74.00)	.764
Race (%)	White	490 (81.1)	2362 (78.2)	.267	250 (79.6)	1217 (77.5)	.7	240 (82.8)	1157 (79.8)	.4801
	Black	55 (9.1)	328 (10.9)		29 (9.2)	165 (10.5)		26 (9.0)	144 (9.9)	
	Other	59 (9.7)	330 (10.9)		35 (11.1)	188 (11.9)		24 (8.2)	149 (10.3)	
Pathological subtype (%)	FA	309 (51.2)	1545 (51.2)	.6	176 (56.1)	876 (55.8)	.169	133 (45.9)	678 (46.8)	.94
	OX	191 (31.6)	977 (32.4)		86 (27.4)	473 (30.1)		105 (36.2)	509 (35.1)	
	FAMI	70 (11.6)	334 (11.1)		28 (8.9)	124 (7.9)		42 (14.5)	204 (14.1)	
	FAT	10 (1.7)	29 (1.0)		9 (2.9)	18 (1.1)		1 (0.3)	11 (0.8)	
	FAWD	24 (4.0)	135 (4.5)		15 (4.8)	79 (5.0)		9 (3.1)	48 (3.3)	
Stage (%)	Localized	437 (72.4)	2083 (69.0)	.04*^c^*	217 (69.1)	1000 (63.7)	.043^*c*^	220 (75.9)	1073 (74.0)	.398
	Regional	130 (21.5)	658 (21.8)		83 (26.4)	441 (28.1)		47 (16.2)	224 (15.4)	
	Distant	37 (6.1)	279 (9.2)		14 (4.5)	129 (8.2)		23 (7.9)	153 (10.6)	
Tumor size, cm (%)	<1	38 (6.3)	151 (5.0)	<.001*^a^*	22 (7.0)	87 (5.5)	.087^*d*^	16 (5.5)	62 (4.3)	<.001*^a^*
	1-2	178 (29.5)	674 (22.3)		90 (28.7)	370 (23.6)		88 (30.3)	294 (20.3)	
	2-4	244 (40.4)	1353 (44.8)		123 (39.2)	724 (46.1)		121 (41.7)	634 (43.7)	
	>4	144 (23.8)	842 (27.9)		79 (25.2)	389 (24.8)		65 (22.4)	460 (31.7)	
Extension (%)	Localized	515 (85.3)	2501 (82.8)	.036*^c^*	278 (88.5)	1319 (84.0)	.024^*c*^	237 (81.7)	1181 (81.4)	.626
	Beyond capsule	73 (12.1)	366 (12.1)		31 (9.9)	175 (11.1)		42 (14.5)	196 (13.5)	
	Further	16 (2.6)	153 (5.1)		5 (1.6)	76 (4.8)		11 (3.8)	73 (5.0)	
LN Met (%)	Yes	32 (5.3)	209 (6.9)	.17	15 (4.8)	109 (6.9)	.198	17 (5.9)	106 (7.3)	.451
	No	572 (94.7)	2811 (93.1)		299 (95.2)	1461 (93.1)		273 (94.1)	1344 (92.7)	
Dis Met (%)	Yes	37 (6.1)	276 (9.1)	.02*^c^*	14 (4.5)	127 (8.1)	.034*^c^*	23 (7.9)	152 (10.5)	.226
	No	567 (93.9)	2744 (90.9)		300 (95.5)	1443 (91.9)		267 (92.1)	1298 (89.5)	
Multifocal (%)	Yes	66 (18.4)	287 (16.0)	.534	24 (18.9)	97 (6.2)	.595	42 (14.5)	190 (13.1)	.813
	No	292 (81.6)	1503 (84.0)		103 (81.1)	538 (34.3)		189 (65.2)	965 (66.6)	
TC-specific mortality (%)	Yes	35 (5.8)	255 (8.4)	.035*^c^*	13 (4.1)	130 (8.3)	.016^*c*^	22 (7.6)	122 (8.4)	.726
	No	569 (94.2)	2765 (91.6)		301 (95.9)	1440 (91.7)		268 (92.4)	1328 (91.6)	
Marital status (%)	Married	352 (58.3)	1759 (58.2)	≥.999	191 (60.8)	982 (62.5)	.61	161 (55.5)	789 (54.4)	.78
	Unmarried	252 (41.7)	1261 (41.8)		123 (39.2)	588 (37.5)		129 (44.5)	661 (45.6)	
Insurance (%)	Yes	269 (44.5)	1332 (44.1)		72 (22.9)	360 (22.9)		197 (67.9)	974 (67.2)	
	No	3 (0.5)	28 (0.9)	.574	2 (0.6)	10 (0.6)	≥.999	1 (0.3)	16 (1.1)	.486
	Not 2007+	332 (55.0)	1660 (55.0)		240 (76.4)	1200 (76.4)		92 (31.7)	460 (31.7)	
Education (median [IQR])		83.55 (79.18-88.68)	84.36 (79.46-88.42)	.217	83.55 (78.36-86.43)	83.55 (79.46-87.52)	.032*^c^*	85.40 (79.21-89.65)	86.00 (80.27-89.27)	.746
Income (median [IQR])		5525.00 (4227.00-6102.00)	5548.00 (4244.00-6191.75)	.315	5087.00 (4219.00-5683.98)	5316.00 (4219.00-5829.00)	.248	5607.50 (4555.25-6387.50)	5620.00 (4586.25-6799.50)	.363
Unemployment (median [IQR])		7.75 (5.69-8.70)	7.68 (5.59-8.70)	.972	7.53 (5.30-8.23)	7.39 (5.25-8.23)	.834	7.80 (5.94-9.60)	7.80 (6.03-9.53)	.875

Abbreviations: BC, breast cancer; BC-1st, TC patients diagnosed after BC; Dis, distant; FA, follicular adenocarcinoma, NOS; FAT, FA trabecular; FAWD, FA well differentiated; IQR, interquartile range; LN, lymph node; Met, metastases; OX, oxyphilic adenocarcinoma; TC, thyroid cancer; TC-1st, TC patients diagnosed before BC; TCnoBC, TC patients without history of BC.

^*a*^ *P* less than .001; *^b^P* less than .01; *^c^P* less than .05; *^d^P* less than .1.

Pathophysiological types, LN metastasis, and multifocality of PTC or FTC showed no association with a history of BC (all *P*> .05). SES factors of marital status, median family income, and unemployment rate were not different between TC-BC and TCnoBC patients (see Tables 1 and 2). Among PTC, TC-BC and BC-1st patients included a higher proportion of White individuals and higher rates of health-care insurance and at-least high school education, but not TC-1st patients (see Table 1). In FTC, except for TC-1st patients who had a different education level compared with matched TCnoBC patients, there was no difference in other parameters when comparing groups (see Table 2). SES and demographic factors showing difference across groups were treated as variables to be controlled in multivariable analyses.

### Relationship Between Thyroid Cancer-specific Cumulative Mortality/Survival and History of Breast Cancer

**Figure 1.**
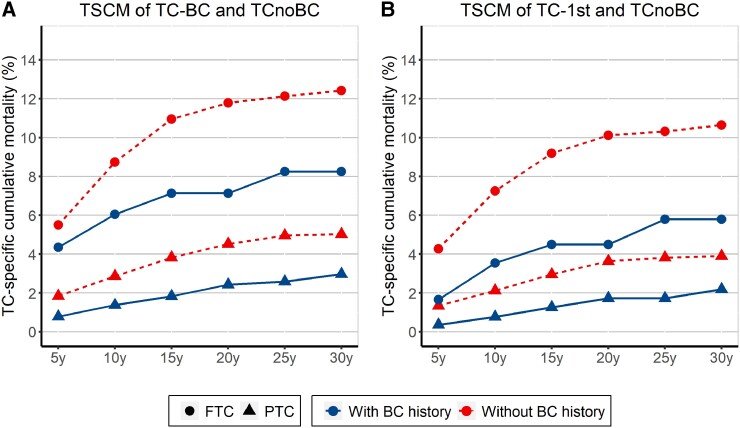
Thyroid cancer (TC)-specific cumulative mortality (TSCM) at 5, 10, 15, 20, 25, and 30 years in TC patients with or without a history of breast cancer (BC). TSCM in A, all TC patients with a history of BC (TC-BC) and in B, TC patients whose TC was diagnosed before BC (TC-1st) compared with those without a history of BC (TCnoBC) in papillary TC (PTC) and follicular TC (FTC), respectively. The circular dotted line represents FTC, and the triangular dotted line represents PTC. The solid curve represents TC with a history of BC, and the dashed curve represents TC without a history of BC.

The comparisons of TSCM at 5 years to 30 years between TC patients with and without a history of BC in PTC or FTC are shown in Fig. 1 (TC-BC/TC-1st), Supplementary Fig. S2 (BC-1st), and Supplementary Table S1 in the supplementary materials (13). For PTC, the TSCMs were 0.78% vs 1.84%, 1.37% vs 2.86%, 2.42% vs 4.52%, and 2.97% vs 5.02% in TC-BC vs the matched TCnoBC patients at 5 years, 10 years, 20 years, and 30 years, respectively (*P* < .001). When cancer occurrence sequence was considered, lower TSCMs were seen both in TC-1st (*P* < .001) and BC-1st (*P* = .001) patients compared with their correspondingly matched TCnoBC patients. However, TSCMs were more prominently different between TC-1st and matched TCnoBC patients, being 0.36% vs 1.34%, 0.77% vs 2.12%, and 1.72% vs 3.63% at 5, 10, and 20 years, respectively. For FTC, reduced TSCMs were observed in TC-BC (*P* = .02) and TC-1st (*P* = .01) patients compared with matched TCnoBC patients, respectively; TSCMs of FTC were more clearly lower in FTC-1st patients compared with matched TCnoBC patients, being 1.66% vs 4.27%, 3.54% vs 7.25%, and 4.49% vs 10.11% at 5, 10, and 20 years, respectively (*P* = .001). No statistically significant difference in TSCM of FTC was found between BC-1st and matched TCnoBC patients (*P* = .634).

**Figure 2.**
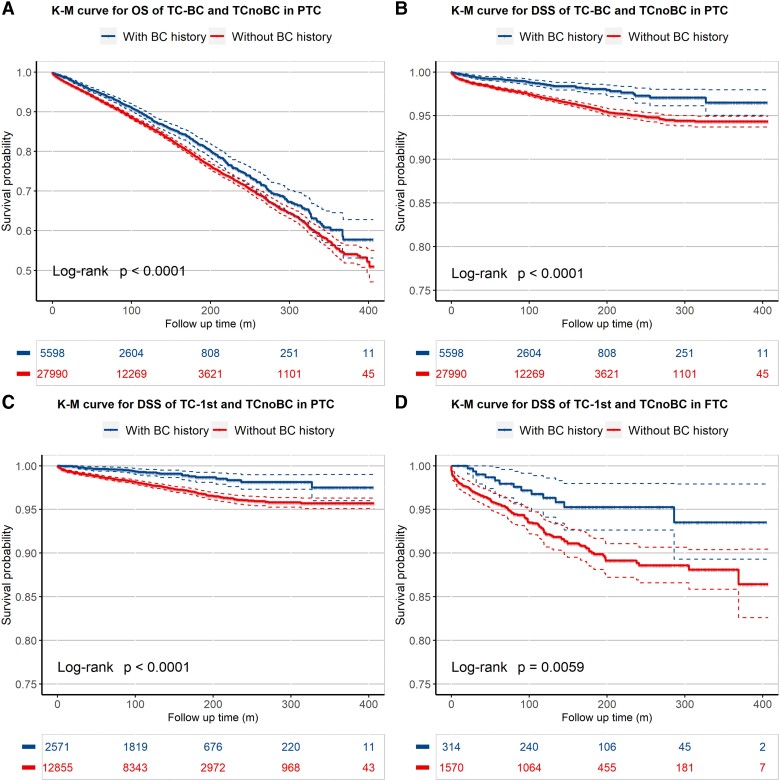
Kaplan-Meier analysis of the effects of a history of breast cancer (BC) on thyroid cancer (TC)-specific and overall survivals of patients in various settings. The protective effects of a history of BC on the prognosis of TC are revealed in the A, overall survival and B, TC-specific survival of all papillary TC (PTC) patients with a history of BC; C, the TC-specific survival of PTC patients whose TC was diagnosed before BC; and D, the TC-specific survival of follicular TC (FTC) patients whose TC was diagnosed before BC. The blue curve represents TC with a history of BC, and the red curve represents TC without a history of BC. The solid curve represents the survival probability over time in the Kaplan-Meier analysis and the dashed curve represents its 95% CIs.

K-M analyses revealed that TC-BC patients had a survival advantage on both overall and PTC-specific survivals than matched TCnoBC patients (Fig. 2A and 2B) (both *P* < .001). This pattern was particularly prominent in the comparison of overall (Supplementary Fig. S3A in the supplementary materials (13)) and PTC-specific (Fig. 2C) survival between TC-1st and TCnoBC patients (both *P* < .001). In comparison with TCnoBC patients, BC-1st patients did not show a difference in overall survival (*P* = .660; Supplementary Fig. S3B) but did show a better PTC-specific survival (*P* = .001; Supplementary Fig. S3C) (see supplementary materials (13)). For FTC, K-M analysis showed a slower decline in the FTC-specific survival curve (*P* = .018; Supplementary Fig. S3E) but not the overall survival curve (*P* = .17; Supplementary Fig. S3D) in TC-BC patients compared with the matched TCnoBC (see supplementary materials (13)). When the cancer occurrence sequence was considered, K-M analysis showed a slower decline of FTC-specific (*P* = .006; Fig. 2D) and overall (*P* = .016; Supplementary Fig. S3F) survival curves in TC-1st patients compared with the matched TCnoBC patients; FTC in BC-1st patients, however, did not show a difference in overall or FTC-specific survival curve on K-M analysis (both *P* > .05; Supplementary Figs. S3G and 3H) (see supplementary materials (13)).

On Cox regression analysis (Table 3), PTC patients of TC-BC (*P* < .001), TC-1st (*P* < .001), and BC-1st (*P* = .002) all had a lower risk of TC-specific mortality compared with their correspondingly matched TCnoBC patients in the univariable model, which remained significant after adjustment for SES factors of race, medical insurance, and education level (both *P* < .001 in TC-BC and TC-1st; *P* = .003 in BC-1st patients). When adding PTC tumor characteristics of tumor size, distant metastasis, and extension to the multivariable model, TC-BC and TC-1st patients still showed a lower TC-specific mortality risk (both *P* < .001), but BC-1st patients did not (*P* = .153). TC-1st patients showed a particularly decreased TC-specific mortality, with HRs of 0.397 (95% CI, 0.268-0.588) in the univariable model, 0.399 (95% CI, 0.270-0.591) in the multivariable model adjusted for SES factors, and 0.409 (95% CI, 0.276-0.607) in the multivariable model adjusted for SES factors and tumor characteristics. For FTC, there was a lower TC-specific mortality risk in TC-BC (*P* = .019) and TC-1st patients (*P* = .007) but not BC-1st patients (*P* = .741) using the univariable model. This pattern was also seen in the multivariable model adjusted for SES factors (TC-BC, *P* = .016; TC-1st, *P* = .006; BC-1st, *P* = .789) but not in multivariable adjustment for both SES factors and tumor characteristics (all *P* > .05), except for an HR of 0.568 (95% CI, 0.319-1.011) for TC-1st vs TCnoBC, showing a marginal significance (*P* = .054).

**Table 3.** Effects of history of breast cancer on thyroid cancer-specific mortality in Cox regression models

Comparison		Univariable model			Multivariable model*^e^*			Multivariable model*^f^*		
		HR	95% CI	*P*	HR	95% CI	*P*	HR	95% CI	*P*
PTC	TC-BC vs TCnoBC	0.472	0.370-0.601	<.001*^a^*	0.477	0.375-0.608	<.001*^a^*	0.529	0.415-0.675	<.001*^a^*
	TC-1st vs TCnoBC	0.397	0.268-0.588	<.001*^a^*	0.399	0.270-0.591	<.001*^a^*	0.409	0.276-0.607	<.001*^a^*
	BC-1st vs TCnoBC	0.607	0.445-0.827	.002*^b^*	0.625	0.458-0.852	<.001*^b^*	0.797	0.584-1.088	.153
FTC	TC-BC vs TCnoBC	0.656	0.461-0.934	.019*^c^*	0.648	0.455-0.922	.016*^c^*	0.799	0.560-1.140	.215
	TC-1st vs TCnoBC	0.458	0.259-0.810	.007*^b^*	0.448	0.253-0.792	.006*^b^*	0.568	0.319-1.011	.054^*d*^
	BC-1st vs TCnoBC	0.926	0.588-1.459	.741	0.940	0.597-1.480	.789	1.262	0.795-2.003	.324

Abbreviations: BC, breast cancer; BC-1st, TC patients diagnosed after BC; CI, confidence interval; FTC, follicular TC; HR, hazard ratio; PTC, papillary TC; SEER, Surveillance, Epidemiology and End Results; SES, socioeconomic status; TC, thyroid cancer; TC-1st, TC patients diagnosed before BC; TCnoBC, TC patients without history of BC.

*^a^P* less than .001; *^b^P* less than .01; *^c^P* less than .05; *^d^P* less than .1.

^*e*^Multivariable model adjusted by SES factors including race, medical insurance, education levels.

^*f*^Multivariable model adjusted by SES factors including race, medical insurance, and education levels and clinicopathological characteristics including SEER stage, tumor size, and distant metastasis and extension.

### Relationship Between Estrogen/Progesterone Receptor Status in Breast Cancer and Thyroid Cancer-specific Patient Survival

TC-specific survival of patients with a history of ER-positive vs ER-negative and PR-positive vs PR-negative BC in various comparison settings are presented in Table 4. No effect of ER or PR status in BC on TC-specific survival was found in the comparisons in FTC patients on the 3 models (all *P* > .05). In PTC, in either the univariable or the SES-adjusted multivariate model, no significant difference was found in the comparisons (all *P* > .05). However, when dissected by tumor occurrence sequence, in the group of TC-1st patients, a marginal significance in the PTC-specific mortality reduction was observed in patients with ER-positive BC vs ER-negative BC (HR 0.433; 95% CI, 0.1692-1.106; *P* = .08), which became significant on multivariable adjustment for both SES factors and tumor characteristics (HR 0.326; 95% CI, 0.127-0.834; *P* = .019). These results suggested that a history of ER-positive status in BC might have a protective effect on TC-specific survival, particularly in TC-1st patients.

**Table 4.** Effects of history of breast cancer on thyroid cancer-specific mortality by different estrogen receptor/progesterone receptor status in Cox regression models

Comparison			Univariable model			Multivariable model*^e^*			Multivariable model*^f^*		
			HR	95% CI	*P*	HR	95% CI	*P*	HR	95% CI	*P*
PTC	TC-BC	ER + vs ER−	0.738	0.402-1.358	.329	0.739	0.401-1.363	.333	0.617	0.333-1.144	.125
		PR + vs PR−	0.951	0.546-1.655	.858	0.948	0.543-1.653	.849	0.842	0.480-1.477	.549
	TC-1st	ER + vs ER−	0.433	0.169-1.106	.080*^d^*	0.439	0.171-1.127	.087*^d^*	0.326	0.127-0.834	.019*^c^*
		PR + vs PR−	0.557	0.234-1.329	.187	0.550	0.231-1.314	.179	0.488	0.204-1.166	.106
	BC-1st	ER + vs ER−	1.154	0.512-2.599	.730	1.158	0.512-2.622	.725	0.973	0.426-2.224	.949
		PR + vs PR−	1.411	0.675-2.950	.360	1.412	0.674-2.958	.360	1.178	0.556-2.494	.669
FTC	TC-BC	ER + vs ER−	0.714	0.309-1.653	.432	0.697	0.299-1.624	.403	0.530	0.220-1.279	.158
		PR + vs PR−	0.931	0.431-2.012	.856	0.902	0.416-1.954	.793	0.953	0.420-2.158	.907
	TC-1st	ER + vs ER−	0.799	0.172-3.721	.775	0.671	0.140-3.220	.618	0.651	0.123-3.444	.614
		PR + vs PR−	0.642	0.188-2.195	.480	0.439	0.123-1.565	.204	0.397	0.101-1.553	.184
	BC-1st	ER + vs ER−	0.760	0.278-2.075	.593	0.715	0.260-1.967	.516	0.433	0.146-1.284	.131
		PR + vs PR−	1.173	0.430-3.203	.755	1.036	0.373-2.879	.946	0.931	0.302-2.867	.901

Abbreviations: BC, breast cancer; BC-1st, TC patients diagnosed after BC; CI, confidence interval; ER, estrogen receptor; FTC, follicular TC; HR, hazard ratio; PR, progesterone receptor; PTC, papillary TC; SEER, Surveillance, Epidemiology and End Results; SES, socioeconomic status; TC, thyroid cancer; TC-1st, TC patients diagnosed before BC.

*^a^P* less than .001; *^b^P* less than .01; *^c^P* less than .05; *^d^P* less than .1.

^*e*^Multivariable model adjusted by SES factors including race, medical insurance, education levels.

^*f*^Multivariable model adjusted by SES factors including race, medical insurance, and education levels and clinicopathological characteristics including SEER stage, tumor size, and distant metastasis and extension.

## Discussion

Previous studies have established an intrinsic association between TC and BC (4, 7, 8) and also an association between a history of TC and lower BC-specific mortality (12). These studies suggest that occurrence both of TC and BC in the same patient may represent a special disease entity with unique clinical outcomes. It has not yet been defined whether a history of BC may affect the prognosis of TC. We demonstrate here that a history of BC indeed has a profound protective effect on TC-specific patient survival both in PTC and FTC, albeit more robust in the former. This effect in PTC was seen regardless of the occurrence sequences of TC and BC, but with more evident in TC-1st patients. In FTC, the effect was largely restricted to TC-1st patients. We are unable to explain mechanistically why TC-1st patients had a better prognosis. One reason could be that younger age seems to be associated with a better protective effect of a history of BC on the prognosis of TC as patients were younger at the diagnosis of TC in TC-1st patients than in BC-1st patients (age 55.00 years [45.00-66.00 years] vs 67.00 years [57.00-74.00 years]) (see Table 2).

Several studies investigated the relationship between a history of BC and clinical outcomes of TC (4, 19, 20) with inconsistent and even contradictory conclusions. Inconsistent control of confounding factors, such as TC types, patient age and SES factors, and TC incidence densities may be a cause. The present study was designed to minimize the effects of such confounders. To this end, we separately analyzed PTC and FTC, which did show different patterns in the effect of a history of BC. Patient age at diagnosis of TC and TC incidence densities across years were strictly controlled between the exposure and the control groups using a propensity score matching approach, effectively minimizing the bias caused by the higher patient age at the diagnosis of TC in BC-1st patients and treatment strategy changes over the years. Inclusion of SES factors and clinicopathological characteristics in the Cox regression model largely reduced their potential confounding bias. With these measures, we found dramatically decreased mortality both in TC-1st and BC-1st patients, especially in PTC, which differed from the somewhat conflicting results between TC-1st and BC-1st in previous studies (4, 19, 20). Also, we included only BC survivors rather than all BC patients to exclude the excess death caused by BC in the TC-BC group and made it more comparable with the TC-only group in view of the well-known low TC-specific mortality and high BC-specific mortality. In this way, the protective effect of a history of BC on TC-specific survival and overall survival could be more accurately evaluated, avoiding yielding the contradictory results between TC-specific and overall survival seen in previous studies (4, 19, 20). On the other hand, by excluding such cases, the behavior and clinical performance of TC that occurred in such patients could not be evaluated. It is likely, however, that TC in such a patient was less aggressive as the patient died of BC unlike in other TC-BC cases, where the patient died of TC.

The bidirectional relationship between TC and BC in their effects on each other's clinical outcomes likely represents a special disease entity with a specific biological basis. A unique genetic background in such patients may be 1 mechanism for the occurrence of the 2 cancers in the same patient. A cohort study of 13 978 females with BC demonstrated that a family history of cancer was an independent predictor for TC in BC patients (21). Partial inheritance of TC and BC occurred in patients with Cowden syndrome (CS) or CS-like diseases (22). These patients harbor germline mutations in the genes of the PI3K/AKT pathway or related signaling pathways, such as *PTEN*, *KLLN*, and *SDHx* (23-27). Other germline mutations in *PARP4*, *CHEK2*, and *MUS81* may also play a role (28-30). Interestingly, CS is more commonly associated with FTC while our results showed that the protective effect of a history of BC was more robust and consistent in PTC than FTC, suggesting the existence of an alternative genetic background, but the small cohort size of FTC in the present study makes the conclusion short of being definitive. The finding in our study of a particularly lower TC-specific mortality in patients with a history of ER-positive BC, especially in PTC-1st patients, is direct evidence suggesting the presence of a common biological basis linking the two cancers to form a unique disease entity. This is further supported by several previous studies that linked TC and BC by showing the tumor-promoting effects of estrogen and progesterone in both cancers (31-34).

## Conclusion

This comparative study on a special cohort of TC patients demonstrates a strong protective effect of a history of BC on TC tumor behavior and TC-specific patient survival. This effect was most robust with PTC in TC-1st patients, particularly in patients with a history of ER-positive BC. Occurrence of both TC and BC in the same patient may represent a special disease entity with a unique underlying biological or, likely, genetic background that needs to be identified. Clinically, the findings in this study may be helpful for more precise prognostication of TC in such patients.

## Data Availability

Original data generated and analyzed during this study are included in this published article or in the data repositories listed in “References.”

## Abbreviations:

BC: breast cancer
BC-1st: TC patient diagnosed after BC
CI: confidence interval
CS: Cowden syndrome
ER: estrogen receptor
FTC: follicular thyroid cancer
HER2: human epidermal growth factor 2
HR: hazard ratio
IQR: interquartile range
K-M: Kaplan-Meier
LN: lymph node
NA: not available
PR: progesterone receptor
PTC: papillary thyroid cancer
SEER: Surveillance, Epidemiology and End Results
SES: socioeconomic status
TC: thyroid cancer
TC-1st: TC patient diagnosed before BC
TC-BC: thyroid cancer patients with a history of breast cancer
TCnoBC: thyroid cancer patients without a history of breast cancer
TSCM: TC-specific cumulative mortality

## References

[1] Siegel RL, Miller KD, Jemal A. Cancer statistics, 2016. CA Cancer J Clin. 2016;66(1):7‐30. 26742998 10.3322/caac.21332

[2] Mao Y, Xing M. Recent incidences and differential trends of thyroid cancer in the USA. Endocrine Related Cancer. 2016;23(4):313‐322. 26917552 10.1530/ERC-15-0445 PMC4891202

[3] Huang J, Ngai CH, Deng Y, et al Incidence and mortality of thyroid cancer in 50 countries: a joinpoint regression analysis of global trends. Endocrine. 2023;80(2):355‐365. 36607509 10.1007/s12020-022-03274-7

[4] Kuo JH, Chabot JA, Lee JA. Breast cancer in thyroid cancer survivors: an analysis of the surveillance, epidemiology, and end results-9 database. Surgery. 2016;159(1):23‐29. 26522696 10.1016/j.surg.2015.10.009

[5] Haugen BR, Alexander EK, Bible KC, et al 2015 American thyroid association management guidelines for adult patients with thyroid nodules and differentiated thyroid cancer: the American thyroid association guidelines task force on thyroid nodules and differentiated thyroid cancer. Thyroid. 2016;26(1):1‐133. 26462967 10.1089/thy.2015.0020 PMC4739132

[6] Siegel RL, Miller KD, Jemal A. Cancer statistics, 2020. CA Cancer J Clin. 2020;70(1):7‐30. 31912902 10.3322/caac.21590

[7] An JH, Hwangbo Y, Ahn HY, et al A possible association between thyroid cancer and breast cancer. Thyroid. 2015;25(12):1330‐1338. 26442580 10.1089/thy.2014.0561

[8] Subramanian S, Goldstein DP, Parlea L, et al Second primary malignancy risk in thyroid cancer survivors: a systematic review and meta-analysis. Thyroid. 2007;17(12):1277‐1288. 18020916 10.1089/thy.2007.0171

[9] Nielsen SM, White MG, Hong S, et al The breast-thyroid cancer link: a systematic review and meta-analysis. Cancer Epidemiol Biomarkers Prev. 2016;25(2):231‐238. 26908594 10.1158/1055-9965.EPI-15-0833 PMC4770576

[10] Edwards BK, Noone AM, Mariotto AB, et al Annual report to the nation on the status of cancer, 1975–2010, featuring prevalence of comorbidity and impact on survival among persons with lung, colorectal, breast, or prostate cancer. Cancer. 2014;120(9):1290‐1314. 24343171 10.1002/cncr.28509 PMC3999205

[11] Richardson LC, Henley SJ, Miller JW, Massetti G, Thomas CC. Patterns and trends in age-specific black-white differences in breast cancer incidence and mortality—United States, 1999–2014. MMWR Morbidity Mortality Wkly Rep. 2016;65(40):1093‐1098. 10.15585/mmwr.mm6540a1 27736827

[12] Cheng W, Shen X, Xing M. Decreased breast cancer-specific mortality risk in patients with a history of thyroid cancer. PLoS One. 2019;14(10):e0221093. 31644578 10.1371/journal.pone.0221093 PMC6808426

[13] Lin S, Wang Z, Xing M. Supplementary materials for “Association between a history of breast cancer and decreased thyroid cancer-specific mortality”. *Figshare 2023*. Deposited Nov 20, 2023. doi:10.6084/m9.figshare.24588939 PMC11031237 38064679

[14] Fritz A . International Classifcation of Diseases for Oncology, 3rd ed. World Health Organization; 2000.

[15] LeClair K, Bell KJL, Furuya-Kanamori L, Doi SA, Francis DO, Davies L. Evaluation of gender inequity in thyroid cancer diagnosis: differences by sex in US thyroid cancer incidence compared with a meta-analysis of subclinical thyroid cancer rates at autopsy. JAMA Intern Med. 2021;181(10):1351‐1358. 34459841 10.1001/jamainternmed.2021.4804 PMC8406211

[16] Austin PC . An Introduction to propensity score methods for reducing the effects of confounding in observational studies. Multivariate Behav Res. 2011;46(3):399‐424. 21818162 10.1080/00273171.2011.568786 PMC3144483

[17] van Buuren S, Groothuis-Oudshoorn K. mice: Multivariate Imputation by Chained Equations in R. J Stat Softw. 2011;45(3):1‐67.

[18] Ho DE, Imai K, King G, Stuart EA. Matchit: nonparametric preprocessing for parametric causal inference. J Stat Softw. 2011;42(8):1‐28.

[19] Huang J, Huang Y, Zhou L, et al Effect of breast cancer as the first or second primary cancer on the prognosis of women with thyroid cancer: a SEER database analysis. Transl Cancer Res. 2020;9(11):6955‐6962. 35117303 10.21037/tcr-20-2243 PMC8798902

[20] Zhang L, Wu Y, Liu F, Fu L, Tong Z. Characteristics and survival of patients with metachronous or synchronous double primary malignancies: breast and thyroid cancer. Oncotarget. 2016;7(32):52450‐52459. 27223440 10.18632/oncotarget.9547 PMC5239566

[21] Huang NS, Chen XX, Wei WJ, et al Association between breast cancer and thyroid cancer: a study based on 13 978 patients with breast cancer. Cancer Med. 2018;7(12):6393‐6400. 30480382 10.1002/cam4.1856 PMC6308067

[22] Starink TM, van der Veen JP, Arwert F, et al The Cowden syndrome: a clinical and genetic study in 21 patients. Clin Genet. 1986;29(3):222‐233. 3698331 10.1111/j.1399-0004.1986.tb00816.x

[23] Ngeow J, Sesock K, Eng C. Clinical implications for germline PTEN spectrum disorders. Endocrinol Metab Clin North Am. 2017;46(2):503‐517. 28476234 10.1016/j.ecl.2017.01.013

[24] Ni Y, He X, Chen J, et al Germline SDHx variants modify breast and thyroid cancer risks in Cowden and Cowden-like syndrome via FAD/NAD-dependant destabilization of p53. Hum Mol Genet. 2012;21(2):300‐310. 21979946 10.1093/hmg/ddr459 PMC3276278

[25] Pilarski R, Burt R, Kohlman W, Pho L, Shannon KM, Swisher E. Cowden syndrome and the PTEN hamartoma tumor syndrome: systematic review and revised diagnostic criteria. J Natl Cancer Inst. 2013;105(21):1607‐1616. 24136893 10.1093/jnci/djt277

[26] Tan MH, Mester J, Peterson C, et al A clinical scoring system for selection of patients for PTEN mutation testing is proposed on the basis of a prospective study of 3042 probands. Am J Hum Genet. 2011;88(1):42‐56. 21194675 10.1016/j.ajhg.2010.11.013 PMC3014373

[27] Wang Y, He X, Yu Q, Eng C. Androgen receptor-induced tumor suppressor, KLLN, inhibits breast cancer growth and transcriptionally activates p53/p73–mediated apoptosis in breast carcinomas. Hum Mol Genet. 2013;22(11):2263‐2272. 23418309 10.1093/hmg/ddt077

[28] Ikeda Y, Kiyotani K, Yew PY, et al Germline PARP4 mutations in patients with primary thyroid and breast cancers. Endocrine Related Cancer. 2016;23(3):171‐179. 26699384 10.1530/ERC-15-0359 PMC5152685

[29] Pinheiro M, Lupinacci FCS, Santiago KM, et al Germline mutation in MUS81 resulting in impaired protein stability is associated with familial breast and thyroid cancer. Cancers (Basel). 2020;12(5):1289. 32443704 10.3390/cancers12051289 PMC7281423

[30] Siołek M, Cybulski C, Gąsior-Perczak D, et al CHEK2 mutations and the risk of papillary thyroid cancer. Int J Cancer. 2015;137(3):548‐552. 25583358 10.1002/ijc.29426

[31] Vannucchi G, De Leo S, Perrino M, et al Impact of estrogen and progesterone receptor expression on the clinical and molecular features of papillary thyroid cancer. Eur J Endocrinol. 2015;173(1):29‐36. 25862786 10.1530/EJE-15-0054

[32] Manole D, Schildknecht B, Gosnell B, Adams E, Derwahl M. Estrogen promotes growth of human thyroid tumor cells by different molecular mechanisms. J Clin Endocrinol Metab. 2001;86(3):1072‐1077. 11238488 10.1210/jcem.86.3.7283

[33] Kumar A, Klinge CM, Goldstein RE. Estradiol-induced proliferation of papillary and follicular thyroid cancer cells is mediated by estrogen receptors alpha and beta. Int J Oncol. 2010;36(5):1067‐1080. 20372779 10.3892/ijo_00000588 PMC11968770

[34] Bolf EL, Sprague BL, Carr FE. A linkage between thyroid and breast cancer: a common etiology? Cancer Epidemiol Biomarkers Prev. 2019;28(4):643‐649. 30541751 10.1158/1055-9965.EPI-18-0877

